# High-Speed Rail Tunnel Panoramic Inspection Image Recognition Technology Based on Improved YOLOv5

**DOI:** 10.3390/s23135986

**Published:** 2023-06-28

**Authors:** Yixin Duan, Su Qiu, Weiqi Jin, Taoran Lu, Xingsheng Li

**Affiliations:** MOE Key Laboratory of Optoelectronic Imaging Technology and System, Beijing Institute of Technology, Beijing 100081, China; 3120200527@bit.edu.cn (Y.D.); jinwq@bit.edu.cn (W.J.); 3120210536@bit.edu.cn (T.L.); 3220200434@bit.edu.cn (X.L.)

**Keywords:** railway tunnel, catadioptric panoramic imaging system, YOLOv5, CARAFE, CBAM, loss function

## Abstract

In order to meet the fast and accurate automatic detection requirements of equipment maintenance in railway tunnels in the era of high-speed railways, as well as adapting to the high dynamic, low-illumination imaging environment formed by strong light at the tunnel exit, we propose an automatic inspection solution based on panoramic imaging and object recognition with deep learning. We installed a hyperboloid catadioptric panoramic imaging system on an inspection vehicle to obtain a large field of view as well as to shield the high dynamic phenomena at the tunnel exit, and proposed a YOLOv5-CCFE object detection model based on railway equipment recognition. The experimental results show that the mAP@0.5 value of the YOLOv5-CCFE model reaches 98.6%, and mAP@0.5:0.95 reaches 68.9%. The FPS value is 158, which can meet the automatic inspection requirements of railway tunnel equipment along the line and has high practical application value.

## 1. Introduction

Railway tunnel infrastructure inspection guarantees the safe operation of railways. Traditional equipment inspection is based on manual inspection, which is inefficient and costly. Recently, computer vision [1] technology received considerable attention from researchers. Its performance in image recognition, object detection, and other fields has far exceeded that of traditional pattern recognition methods. Automated intelligent inspection technology can improve inspection efficiency and has become the trend in railway tunnel inspection.

Owing to the short interval time between trains inside the high-speed rail tunnel, the car-mounted automatic image detection device is critical for safer and more stable automatic inspection technologies. To achieve the comprehensive detection of the tunnel wall, a camera with a large field of view (FOV) is required. Currently, scholars are conducting various studies on railway tunnels using onboard machine vision devices and image processing techniques. Janiszewski et al. [2] proposed a method for the rapid photogrammetric reconstruction of tunnels using a 360° camera. They used a 360° camera to capture a 10 m section of exposed rock tunnel from 27 positions in the tunnel at the underground research laboratory of Aalto University in Finland. They used the SfM-MVS photogrammetric technique to reconstruct a 3D model, resulting in significant improvements in speed and accuracy. Gavilán et al. [3], from Spain, developed a tunnel-lining detection system using six high-speed cameras and six laser sensors. This system can simultaneously obtain images of the tunnel lining and its 3D contour, with a depth accuracy of 0.5 mm and a longitudinal and transverse resolution of 1 mm. Toru et al. [4], from Pacific Consultants Co., Ltd., Tokyo, Japan, proposed the MIMM-R tunnel detection system consisting of 20 cameras, which can operate at speeds of 50–70 km/h. When captured with a pixel resolution of 1.5 mm, the system can identify cracks wider than 0.3 mm. Zhou et al. [5] from the China Railway Second Survey and Design Institute put forward a tunnel clearance detection technique based on the 3D laser point cloud. By calculating the distance between the tunnel cross-sectional profile and the tunnel clearance detection frame, combined with the clearance calculation method of the detection frame, the tunnel clearance detection was achieved with a precision of 2 cm. Xue et al. [6] proposed a “mobile tunnel profile measurement” system (MTPM-1), which deployed a new type of rotating camera to track a translating laser target, enabling the fast 3D capture of a 100 m tunnel within 3 min. The maximum measurement error for a tunnel with a diameter of 5.5 m is less than 5 mm. The TS series tunnel detection system [7] designed by Spacrtec GmbH from Germany uses a 360° rotating scanning head to acquire images of the tunnel lining cross-section. The 360° range can reach 100 million pixels, and the distance between adjacent scan lines can reach 2–5 mm. Beijing Mingboruier Railway Technology Inspection Co., Ltd., Beijing, China, made a rapid tunnel inspection system [8] which is equipped with a line scan camera, a laser scanner, GPS, and other devices. It can detect lining surface cracks, water leakage, and tunnel clearance. When operating at a speed of 30 km/h, it can measure cracks with a width of 1.0 mm. However, the analysis methods for tunnel image data and surface defects in this detection system are not satisfactory and mainly rely on manual recognition, which is time consuming and labor intensive. Jiang et al. [9], from China, used imaging devices installed on a high-speed comprehensive inspection train to obtain railway environment videos and generate panoramic images by stitching four images together, enabling the fast intelligent inspection of the railway infrastructure. Wang et al. [10], from the China Academy of Railway Sciences, used a performance imaging detection system based on a mobile platform to detect cracks in railway tunnels, achieving a detection rate of 92.6% for cracks wider than 0.3 mm. Attardl et al. [11], from the University of Malta, introduced a visual inspection system in Europe called TInspect, which can install track detection above tunnels. It captures images in the tunnel using low-cost cameras and performs image processing and analysis to determine tunnel defects. This system has high sensitivity and accuracy, but the single wide-angle lens can only provide images of a limited area in the tunnel, requiring multiple cameras or changing camera position to cover the whole tunnel.

These studies commonly employ camera rotation, multi-camera multi-angle shooting, and subsequent image stitching to obtain panoramic images of the tunnel interior. However, such methods lack real-time capabilities and are unable to achieve the real-time acquisition of panoramic image information. Additionally, during the process of single-camera rotation or multi-camera placement, there is a risk of losing images from certain field-of-view angles, resulting in incomplete information retrieval. The presence of multiple lenses in multi-camera systems leads to a larger overall volume and higher manufacturing costs. Furthermore, the large amount of data generated by stitching multiple images reduces image-processing efficiency and imposes high hardware requirements on the system. Moreover, the overall lighting in the tunnel is dim, and the bright light near the tunnel entrance poses challenges for high dynamic and low-illumination scenes for such onboard imaging detection devices. This paper proposes the application of a hyperbolic mirror reflection imaging system in tunnel inspection work. This system has a simple structure and can achieve real-time, wide-field panoramic imaging. Compared to other panoramic imaging systems, the reflection imaging system exhibits higher image quality and less distortion, thereby improving image clarity and accuracy. Consequently, it enhances image-processing efficiency, reduces the use of computational resources, and lowers system costs. Importantly, the system has the capability of high-dynamic-range (HDR) imaging within the tunnel. By placing the system horizontally in front of the inspection vehicle with the bottom end of the reflective mirror facing the bright light at the tunnel exit, the camera sensor captures images reflected by the hyperbolic mirror from the front side of the tunnel, thus physically shielding the bright light at the tunnel exit. Furthermore, active illumination can be employed to achieve clear panoramic imaging of the tunnel walls.

The detection of railway tunnel defects includes cracks, leaking water, surface defects, firmware damage, and misbehavior of signs and other instruments. Traditional image detection methods are based on feature extraction, which recognizes objects by extracting the low-level features of images (such as edges, corners, textures, etc.). Representative algorithms include SIFT, SURF, HOG, etc. However, these algorithms are difficult to adapt to fast detection on moving platforms. Otherwise, the detection speed and detection FOV (resolution) are difficult to trade off, resulting in low detection efficiency. Recently, object recognition methods based on deep learning have begun to be applied to railway detection. Byunghyun Kim et al. [12] used Mask R-CNN to detect the surface of tunnels, extracted cracks, and quantified them using morphological operations. Yang Xu et al. [13] used Faster R-CNN to identify and detect seismic damage to tunnel concrete columns after a severe earthquake. Based on a small-batch stochastic gradient descent algorithm, Yang introduced a four-step alternating training method to automatically identify and locate multiple types of seismic-damaged tunnels. Although Yang’s method has high accuracy, the dataset is relatively simple and cannot represent the actual complex and varied situations. Li Dawei [14] designed a subway tunnel surface automatic detection system, which improved the algorithm’s performance in surface defect detection by proposing a multilayer feature fusion network based on Faster R-CNN and achieved the high-precision identification of defects. J. Chen et al. [15] studied a firmware defect detection method based on deep convolutional networks. They used SSD to locate and detect firmware as well as YOLOv3 for recognition, and then classified the defects into different categories. However, the network model is not end-to-end; thus, it is difficult to implement it. Z. Xing et al. [16] addressed the problem of low contrast between train numbers and train bodies caused by insufficient lighting in tunnels and indoor environments. They used an improved single-scale Retinex algorithm based on brightness control for image enhancement. Additionally, they proposed a train number location algorithm based on speed-up robust features and stroke width transform to accurately locate the train numbers. Z. Xing et al. [17] introduced a deep-learning-based method for identifying wheel tread defects. They enhanced the YOLOv3 algorithm by adding a scale convolutional layer feature pyramid and optimizing the loss function. This improved algorithm can effectively detect defects and faults on the surface of the train wheels.

In summary, the current detection equipment for high-speed rail lines and tunnels has the following issues: With the increasing electrification and automation of high-speed rail in recent years, the number and types of equipment along the railway have significantly increased. Along the high-speed rail lines, the powerful air flow caused by high-speed trains poses a risk of equipment damage or displacement. There is no practical detection system for rapid inspections of this kind. The panoramic detection methods, such as multi-camera stitching images, have drawbacks, such as image stitching errors and slow processing speed, which impose significant difficulties on the acquisition, storage, and processing of images in the system. While laser radar detection can obtain three-dimensional images of the targets along the line, the point cloud is not intuitive enough for users and does not meet the needs of railway operators. Therefore, it is necessary to develop a detection algorithm that is structurally simple, has stable detection performance, can adapt to high inspection speeds, and is user friendly. Therefore, we adopted a catadioptric panoramic imaging system for inspection work; the detected images are large FOV panoramic images yet exhibit certain distortions and non-uniformity. Additionally, in poor weather conditions along the railway or in tunnel scenes, image blurring and low contrast can occur due to insufficient ambient lighting. Furthermore, the size of the target varies significantly when capturing images on a moving train. The traditional YOLOv5 method is ineffective in recognizing these images and is prone to missed detections or false detections. To address this, we propose improvements based on the YOLOv5 algorithm to enhance model performance.

Consequently, we propose a high-speed rail tunnel circumferential inspection image recognition technology based on improved YOLOv5. The main contributions are as follows: (1) The adoption of a catadioptric panoramic imaging system for high-speed rail inspections enables stable, real-time panoramic imaging for railway tunnel inspections. (2) In terms of the algorithm, the content-aware reassembly of features (CARAFE) module is used to replace the original upsampling method, which increases the model’s receptive field and improves the efficiency of aggregating contextual information in a large receptive field. (3) The convolutional block attention module (CBAM) is introduced in the C3 module of the backbone network to emphasize key features and suppress other unimportant information in the image, thus enhancing the model’s robustness to changes in the target scale. (4) The original CIOU loss function is replaced by Focal-EIOU to balance positive and negative samples and improve the convergence speed of the model.

## 2. Materials and Methods

### 2.1. Hyperbolic Reflective Panoramic Imaging System

**Figure 1:**
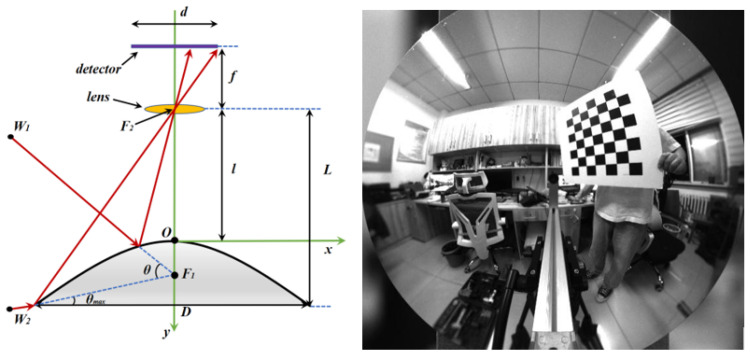
Schematic diagram of single-viewpoint imaging.

As shown in Figure 1, the structure of the catadioptric panoramic imaging system is relatively simple in structure with low light energy loss. It uses a conical reflective mirror to reflect the surrounding horizontal 360° range of objects onto the imaging sensor, thus obtaining a real-time panoramic view of the horizontal FOV. Compared with traditional multi-camera stitching panoramic imaging systems and fisheye lens panoramic imaging systems, the catadioptric panoramic imaging system has significant advantages in system size, structural complexity, and real-time performance [18]. Therefore, it was recently employed in a wide range of application prospects in various fields, such as robot navigation, moving target detection, virtual reality scenes, and video surveillance. Currently, the widely used panoramic vision systems are single-view imaging systems, where the extension lines of each incident ray reflected by the mirror intersect at the same point as shown in Figure 1. This system uses a pinhole imaging method for analysis, which has a simple object–image mapping relationship, and based on the constraint conditions of single-view imaging, the specific surface shape of the mirror can be calculated.

Accordingly, we designed a single-viewpoint catadioptric hyperbolic mirror panoramic vision system using a combination of a hyperbolic mirror and conventional imaging lens. Because the lower focal point of the hyperbolic mirror and the focal point of the perspective camera lens are overlapped, according to the mathematical properties of the hyperbola, incident light directed towards the upper focal point of the hyperbolic mirror reflects towards its lower focal point, thereby allowing the position information of all spatial points on the hyperbolic mirror to be perceived in real time on the image plane. This method is an efficient and reliable way to acquire information in scenarios with high real-time processing requirements. Additionally, the use of this system can effectively avoid high-dynamic-range imaging caused by strong light at tunnel exits, while simultaneously obtaining a large FOV panoramic image, providing convenient conditions for subsequent target recognition.

**Figure 2:**
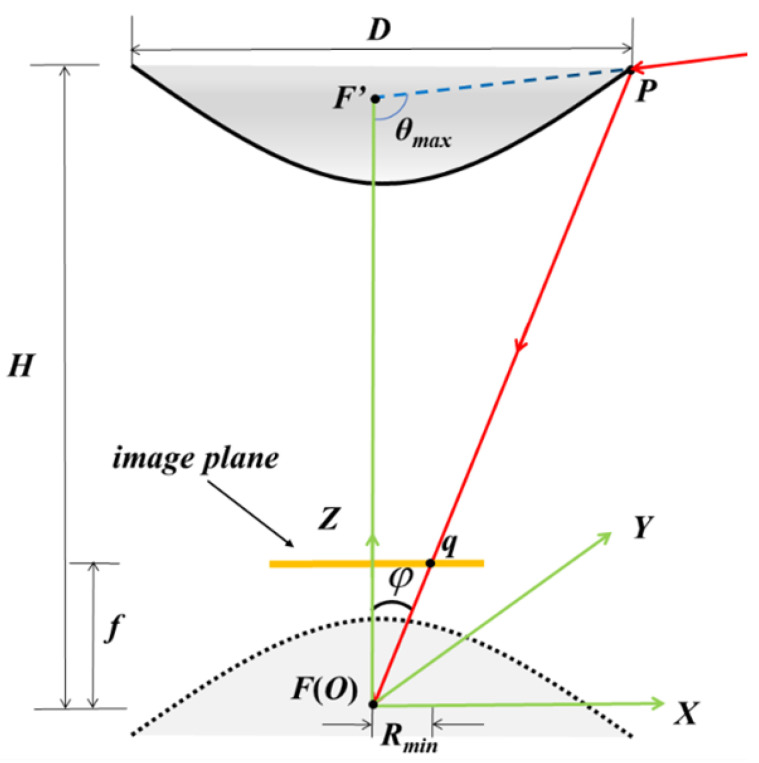
Schematic diagram of the catadioptric imaging system.

The two key parameters of the catadioptric panoramic imaging system are the distance *H* between the top of the reflection mirror and the lens, and the diameter *D* of the bottom edge of the reflection mirror. As shown in Figure 2, the method for calculating the parameters of the hyperbolic reflection mirror is obtained based on geometric analysis:

(1) $$\tan\varphi =\frac{R_{\min}}{f}=\frac{D}{2H}$$

(2) $$\begin{array}{c} a^{2}=\frac{1}{16}\left(4H^{2}+2D^{2}+D^{2}\cot^{2}\left(\theta_{\max}\right)\right)-\frac{D}{8\sin(\theta_{\max})}\sqrt{4H^{2}+D^{2}}\\ b^{2}=\frac{1}{8}D\left(2H\cot\left(\theta_{\max}\right)-D\right)-\frac{D}{8\sin(\theta_{\max})}\sqrt{4H^{2}+D^{2}} \end{array}$$

$R_{min}$ is the distance from the endpoint of the effective pixels on the short side of the image plane rectangle to the principal point of the optical axis, *f* is the focal length of the lens, φ is the camera FOV angle, *a* and *b* are the parameters of the hyperbola, and $\theta_{max}$ is the maximum vertical FOV angle of the system. The values of the quadratic surface reflection mirror parameters *a* and *b* can be obtained through Equations (1) and (2).

**Figure 3:**
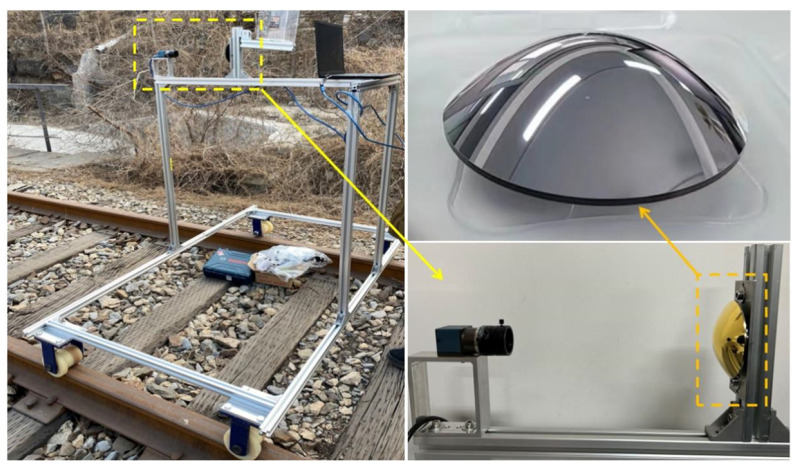
Physical picture of the inspection test vehicle.

Our system includes a Daheng Crystal industrial camera with a focal length of 16 mm, a 2/3 inch CMOS sensor, a pixel size of 3.45 µm, and a resolution of 2448 × 2048. The camera is calibrated using Zhang’s calibration method [19], with resulting parameters of $R_{min}$ = 3.57 mm and *f* = 16.15 mm. The desired distance *H* is 160 mm, which leads to a *D* value of 70.4 mm. The vertical FOV angle $\theta_{max}$ = 90°, and the specific parameters for a hyperbolic mirror are determined to be *a* = 64.31 mm, *b* = 47.58 mm, focal length = 160 mm, and thickness = 15.69 mm. The mirror surface is coated with a silver/gold reflective layer, and its opening diameter is set at 80 mm. The mirror is manufactured according to the particular parameters. The lens and mirror are jointly designed and assembled to ensure complete imaging of the reflected image on the detector. A simple inspection vehicle is constructed using aluminum profiles and equipped with iron rail pulleys for easy image acquisition in the field as shown in Figure 3.

**Figure 4:**
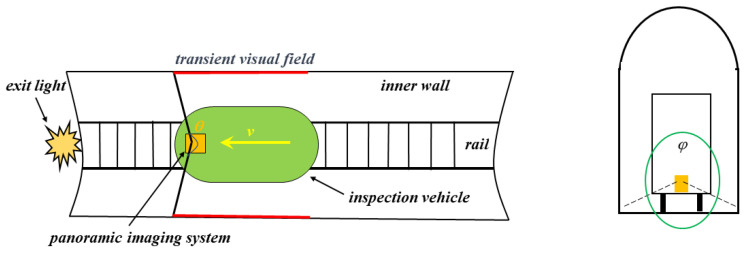
Schematic diagram of the working principle of our system.

The scenario of this system is illustrated in Figure 4. The vehicle-mounted, high-dynamic-range panoramic inspection system is installed at the front of the inspection vehicle, and it performs short-exposure imaging of the cross section of the tunnel’s inner wall along the direction of the train’s forward movement. The horizontal FOV angle θ > 180°, and the vertical FOV angle φ = 360°, which can achieve the panoramic imaging of a large FOV for the tunnel.

**Figure 5:**
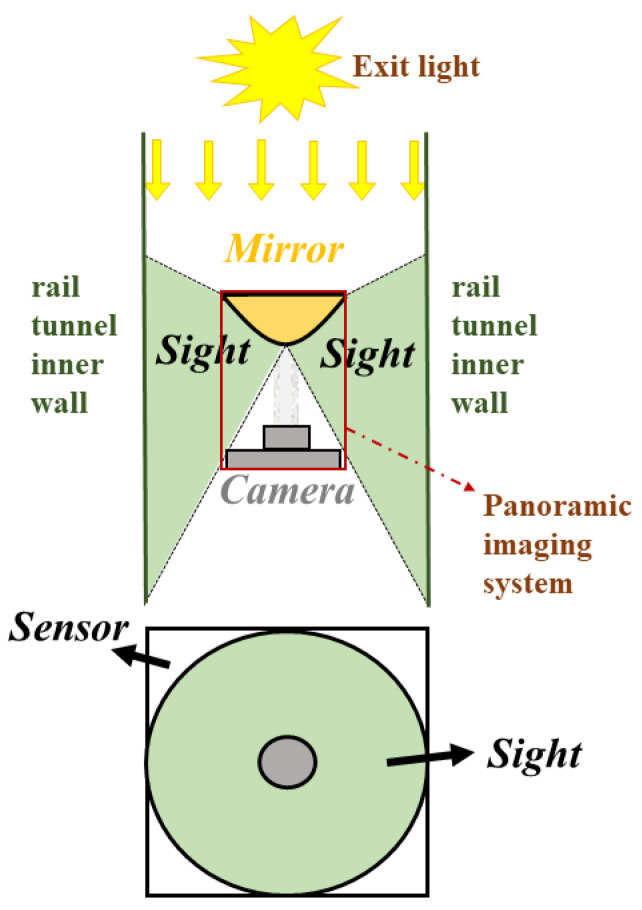
Schematic diagram of catadioptric imaging system shielding the bright light at the tunnel exit.

The schematic diagram of the catadioptric imaging system shielding the bright light at the tunnel exit is shown in Figure 5. The backside of the hyperbolic mirror faces the tunnel exit, and the CMOS sensor in the catadioptric panoramic imaging system can only receive tunnel scenes reflected by the front side of the mirror. Light behind the backside of the mirror, including the bright light at the tunnel exit, is effectively blocked, thereby physically addressing the high dynamic phenomena faced by ordinary industrial cameras or fisheye lenses in tunnels. Then, active illumination is applied to the inner wall of the tunnel to achieve clear panoramic imaging of the inner wall scenes.

**Figure 6:**
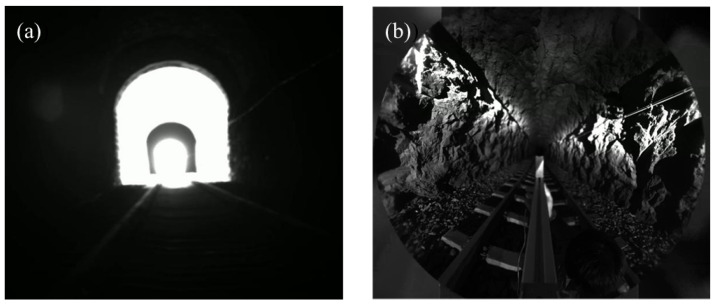
Actual scene of tunnel. (**a**) Tunnel image captured by a normal camera. (**b**) Tunnel image captured by our system.

The actual scene is shown in Figure 6. Figure 6a is an internal scene of the tunnel captured by an ordinary industrial camera. It can be observed that the bright light at the tunnel exit creates a high dynamic and low-illumination environment, and the internal scene information of the tunnel is completely blocked, resulting in poor imaging quality. Figure 6b shows the scene image captured using our system, which has a large imaging FOV and avoids the strong light direction at the tunnel exit, reducing the difficulty of HDR imaging and providing rich image detail information. To compensate for the insufficient illumination of the relatively dark tunnel walls during imaging, we added active near-infrared illumination with the same imaging FOV, which compensates for the lack of illumination and does not affect human visual perception.

### 2.2. YOLOv5

Object detection can be divided into two categories: two-stage and one-stage algorithms. The former extracts image feature maps and then uses region proposals to obtain accurate target and position information, including models, such as R-CNN [20], Fast-RCNN [21], and Faster-RCNN [22]. The latter is one-stage algorithms, which directly detect the entire image to obtain target position and class information based on regression analysis, including the YOLO series [23,24], SSD, etc. In addition, the latest YOLOv7 [25] and various improved algorithms have excellent performance. However, currently, YOLOv5 is still a reliable, stable, and easily deployable and trainable algorithm. Furthermore, it is one of the one-stage detection algorithms with the highest accuracy. Therefore, in this study, we chose YOLOv5 for further improvement.

YOLOv5 is a typical one-stage object detection algorithm proposed by the Ultralytics team in June 2020. Since its inception, it has been continuously updated and iterated. Depending on the depth and width of the network structure, it is divided into several versions of different sizes, such as YOLOv5s, YOLOv5m, YOLOv5l, and YOLOv5x. This study is based on the YOLOv5s version 6.1 for relevant improvements, divided into four parts: the input part, backbone feature extraction network, neck feature fusion network, and output head.

The input part implements image enhancement, data normalization, and data scaling. Among them, data augmentation uses the mosaic augmentation method, which randomly crops, arranges, and splices four different images into a new image, thereby increasing small sample targets and improving the training speed of the network while expanding the dataset.

The backbone network is responsible for extracting general features. The CSPDarknet53 structure is used to reduce computation in inference and lower the memory cost of the model. In YOLOv5 version 6.1, the first layer’s focus structure was changed to a convolutional layer to improve the model’s engineering application speed.

The neck network processes the extracted features to enhance feature diversity, including FPN (feature pyramid network) [26] and PAN (path aggregation network) [27]. FPN conveys significant semantic features from top to bottom, while PAN conveys location features from bottom to top. After passing through multiple layers of the network in FPN, the target information in the lower layers becomes blurry, and the introduction of PAN can compensate, thus enhancing the localization information.

The output head of YOLOv5 adopts the detection head of YOLOv3, where the classification loss and confidence loss use binary cross-entropy (BCE) loss, and the localization loss uses the CIoU loss, which is used to detect the position and category of objects. Common localization loss functions include DIOU loss [28], SIOU loss [29], and EIOU loss [30].

### 2.3. YOLOv5-CCFE Railway Equipment Recognition Algorithm

To conduct automated inspections in railway tunnel scenarios more effectively, we proposed a YOLOv5-CCFE railway equipment recognition algorithm. This algorithm improves the upsampling method in the neck network based on YOLOv5s, incorporated the CBAM attention mechanism [31] into the C3 part of the backbone network, and replaces the original CIOU loss function, achieving good detection results.

#### 2.3.1. Improvement in Upsampling

In the YOLOv5 feature fusion network, the feature enhancement is achieved through the upsampling operation, which enlarges the extracted feature map to obtain a higher resolution image. There are two types of upsampling methods, which are linear interpolation and deconvolution. The former inserts new elements between pixels based on the original image pixels using an appropriate interpolation algorithm, such as the nearest neighbor interpolation, bilinear interpolation, and trilinear interpolation. However, these interpolation methods only consider the sub-pixel neighborhood, resulting in significant discontinuity in the grayscale values of the resampled image, significant loss of image quality, and possible loss of semantic information, leading to inaccurate local features. The latter expands dimensions through convolution. However, deconvolution uses the same convolution kernel for the entire image, which results in poor local change perception ability and increased parameter complexity.

**Figure 7:**
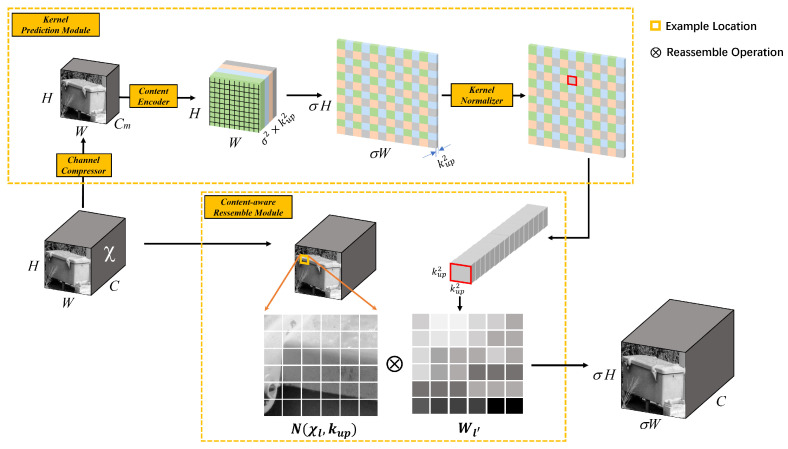
Framework of the CARAFE module.

Consequently, we introduced a content-aware reassembly of features module [32] in the FPN structure to replace the original upsampling module. Compared with traditional upsampling methods, CARAFE has a larger receptive field, which can effectively aggregate contextual information in a large receptive field. It supports content-aware processing for specific instances and can dynamically generate adaptive kernels. Meanwhile, it demands fewer computation resources and is lightweight and easy to integrate. CARAFE consists of an upsampling kernel prediction module and a feature reassembly module as shown in Figure 7. The upsampling kernel prediction module includes three sub-modules: feature map channel compression, content encoder, and upsampling kernel normalizer. Feature map channel compression reduces the number of input feature map channels to **$C_{m}$** using 1 × 1 convolution, which improves the efficiency of CARAFE and significantly reduces the required number of parameters. The content encoder generates a reassembly kernel based on the content of the input feature, which can produce a feature map with a channel number of ***$\sigma^{2}$$k_{up}^{2}$***, where **σ** represents the upsampling rate (usually 2) and **$k_{up}$** represents the upsampling kernel size. To increase the encoder’s receptive field, we added convolution layers of the kernel size during processing to better utilize contextual information in the region. Finally, we normalized each channel of the upsampling kernel using the Softmax function. In the feature reassembly module, we mapped each position in the output feature map back to the input feature map, assumed the center **$k_{up}$** × **$k_{up}$** region and the predicted upsampling kernel for that region, and performed dot product operation to obtain the final output after upsampling.

#### 2.3.2. Introducing CBAM Attention Mechanism

**Figure 8:**
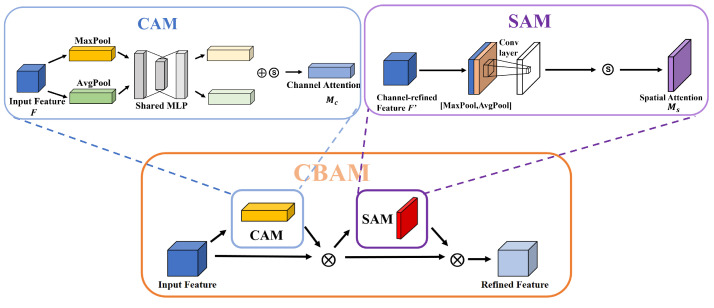
CBAM structure diagram.

In panoramic images along the railway lines, there may be some low-resolution and blurry target objects. Using CBAM, attention regions can be extracted to help the model resist confusing information and focus on useful target objects. CBAM combines the channel attention module (CAM) and spatial attention module (SAM), making it a simple and effective lightweight attention mechanism that can be trained end to end as shown in Figure 8.

The channel attention mechanism compresses input feature maps in the spatial dimension to obtain a one-dimensional vector for further processing. During the compression, both average pooling and max pooling are used to aggregate the spatial information of the feature maps. The results of these pooling operations are then fed into a shared network, combined using element-wise summation, and finally passed through a sigmoid activation function to generate the channel attention map:

(3) $$\begin{array}{c} M_{c}\left(F\right)=\sigma \left(MLP\left(AvgPool\left(F\right)\right)+MLP\left(MaxPool\left(F\right)\right)\right)\\ \;\;\;\;=\sigma (W_{1}\left(W_{0}\left(F_{\mathrm{avg}}^{c}\right)\right)+W_{1}\left(W_{0}\left(F_{\max}^{c}\right)\right)) \end{array}$$

*F* represents the input feature map; σ denotes the sigmoid operation; *MLP* stands for multilayer perceptron; *Avgpool* denotes average pooling; and *Maxpool* denotes max pooling. When performing average pooling on the spatial dimension, each pixel on the feature map receives feedback, while max pooling provides gradient feedback only at the location of the pixel with the highest response in the feature map during gradient backpropagation. Spatial attention compresses the channels by separately applying average pooling and max pooling along the channel dimension. The pooled results are then concatenated and passed through a convolutional layer to obtain the spatial attention map:

(4) $$\begin{array}{c} M_{s}\left(F\right)=\sigma \left(f^{7\times 7}\left(\left[AvgPool\left(F\right);MaxPool\left(F\right)\right]\right)\right)\\ =\sigma (f^{7\times 7}\left(\left[F_{avg}^{s};F_{\max}^{s}\right]\right)) \end{array}$$

*f* denotes convolution and *7 × 7* denotes the size of the convolutional kernel. Lastly, we combine them to obtain

(5) $$\begin{array}{c} y_{1}=M_{c}\left(F\right)⊗F\\ y_{2}=M_{s}\left(y_{1}\right)⊗y_{1} \end{array}$$

*F* represents the input feature map. After applying the channel attention mechanism **$M_{c}$**, the weights are obtained and multiplied with the original feature map, resulting in the channel-attention-modulated feature map **$y_{1}$**. This **$y_{1}$** is then passed through the spatial attention mechanism **$M_{s}$** and multiplied with itself, yielding the final feature map **$y_{2}$**.

**Figure 9:**
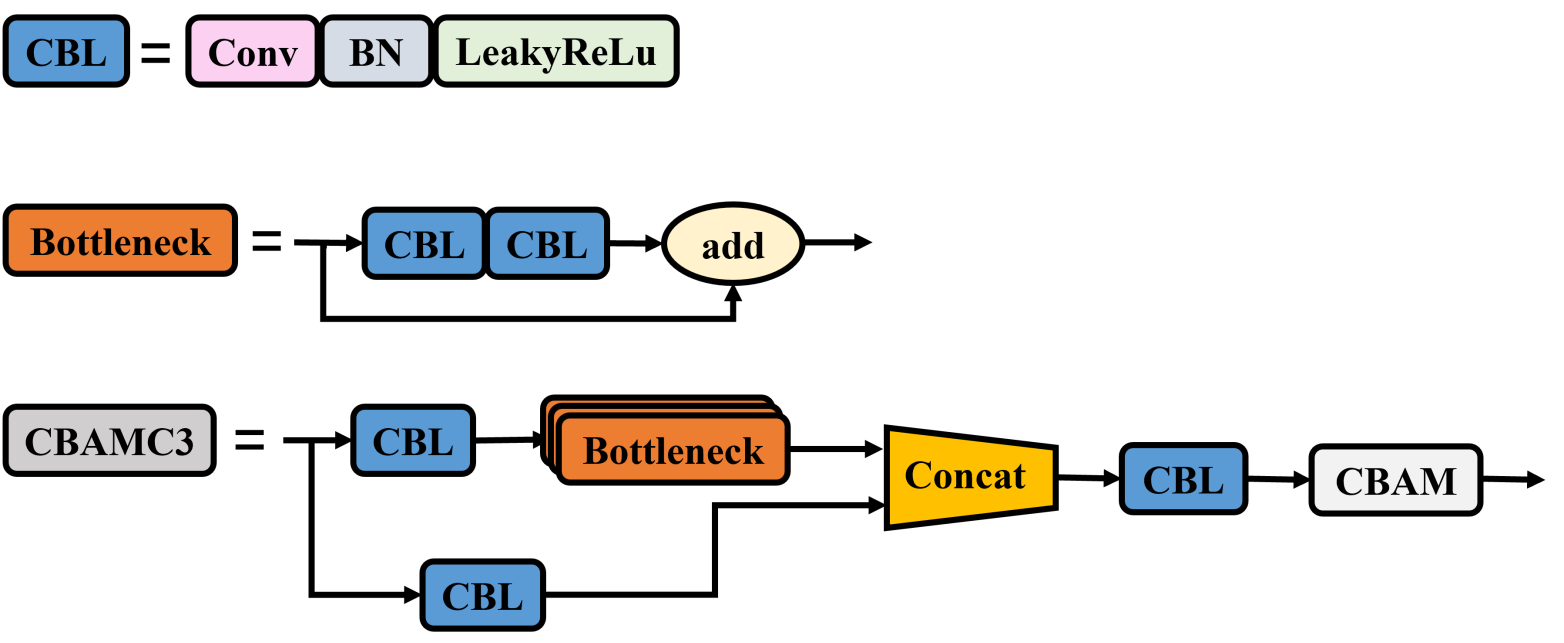
CBAMC3 architecture diagram.

The C3 module in the backbone network of YOLOv5 is a lightweight convolutional neural network module that extracts the features of targets by increasing the depth and receptive field of the network. After the information processing by the C3 module in the backbone network, the feature information is prone to loss. Therefore, our method combines the C3 module in the backbone network with an attention mechanism to improve it and create the CBAMC3 module as shown in Figure 9. The addition of the attention mechanism replaces the standard convolutional module at the end of the C3 module with an attention mechanism to extract features, allowing the model to focus on the features of the target and accurately identify the status of different targets, thereby improving detection performance and reducing false detection rates.

#### 2.3.3. Optimization of the Loss Function

The loss function of the YOLOv5 model includes the following types:

1. Classification loss (cls loss): This loss is used to determine if the model can accurately identify the objects in the image and classify them into the correct category.
2. Confidence loss (obj loss): This loss is used to measure the difference between the predicted and true boxes.
3. Box regression loss (box loss): This loss is used to measure the difference between the predicted boxes and true boxes to accurately localize the objects.

In YOLOv5, the classification loss and the confidence loss are calculated using cross-entropy loss, while the box regression loss is calculated using CIOU. The CIOU loss considers the overlapping area, distance between center points, and aspect ratios, which can be expressed as

(6) $$IOU\left(A,B\right)=\frac{A\cap B}{A\cup B}$$

(7) $$L_{\mathrm{CIOU}}=1-IOU+\frac{\rho^{2}\left(A,B\right)}{C^{2}}+\tau \upsilon$$

(8) $$\upsilon =\frac{4}{\pi^{2}}{\left(\arctan\frac{\omega^{gt}}{h^{gt}}-\arctan\frac{\omega }{h}\right)}^{2}$$

The IOU function is used to measure the overlap between the target and predicted boxes, where *A* represents the region of the target box and *B* represents the region of the predicted box. $L_{CIOU}$ is the loss function of CIOU, where *C* is the diagonal length of the smallest area, $\rho^{2}\left(A,B\right)$ is the Euclidean distance between the centers of *A* and *B*, τ is a positive balancing parameter, and ν is the consistency of the aspect ratio. The CIOU loss slightly reflects the difference in aspect ratio and increases the similarity of the aspect ratio, but it occasionally hinders the true difference between the aspect ratio and confidence, and it does not consider the balance of difficult and easy samples.

In our method, we introduce Focal-EIOU loss to replace the CIOU loss in the original model. Based on the CIOU, the impact factors of the aspect ratio are separated, and the length and width of the target and anchor boxes are calculated separately. This approach retains the characteristics of the CIOU loss while reducing the difference in width and height between the target and anchor boxes, which facilitates the convergence of the model and slightly improves its accuracy [33]. Additionally, the focal loss effectively reduces the contribution of anchor boxes with low overlap with the target box to the bounding box regression, reducing the weight of a large number of simple negative samples during training. This approach focuses on high-quality anchor boxes in the regression process and solves the problem of severe imbalance between positive and negative samples in the actual images collected along the high-speed rail equipment. The specific expression is as follows:

(9) $$L_{\mathrm{EIOU}}=L_{IOU}+L_{dis}+L_{asp}==1-IOU+\frac{\rho^{2}\left(b^{0},b^{gt}\right)}{C^{2}}+\frac{\rho^{2}\left(\omega ,\omega^{gt}\right)}{C_{\omega }^{2}}+\frac{\rho^{2}\left(h,h^{gt}\right)}{C_{h}^{2}}$$

(10) $$L_{\mathrm{Focal}-\mathrm{EIOU}}=IOU^{\gamma }L_{\mathrm{EIOU}}$$

In this equation, **$C_{\omega }^{2}$** and **$C_{h}^{2}$** represent the width and height of the minimum bounding rectangle for the predicted and target boxes, respectively. **$\rho^{2}$** denotes the Euclidean distance between the predicted and target boxes. **$b^{0}$** and **$b^{gt}$** are the center points of the predicted and target boxes, respectively. ω and *h* are the width and height of the predicted box, and **$\omega^{gt}$** and **$h^{gt}$** are the width and height of the target box.

**Figure 10:**
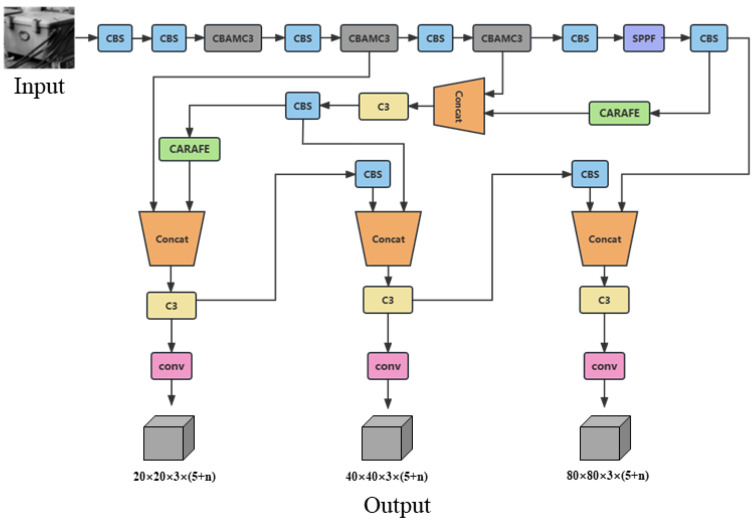
YOLOv5-CCFE architecture diagram.

The overall proposed network architecture diagram is shown in Figure 10. Based on YOLOv5, we introduced the CARAFE module, incorporated the CBAM module into the C3 part of the backbone, and utilized the Focal-EIOU module to enhance the overall performance of the model. For ease of reference in subsequent experiments and discussions, we refer to this model as YOLOv5-CCFE (YOLOv5 + CARAFE + CBAM + FocalEIOU). When inputting an image or video for detection, the YOLOv5-CCFE model outputs three sets of anchor boxes with corresponding confidence scores at different scales. Non-maximum suppression is applied to these anchor boxes to determine the final position and category of the detected objects.

## 3. Experiments and Analysis

### 3.1. Experimental Platform

The experimental devices and parameters are shown in Table 1. The hyperparameters for the data training are epoch = 100, batch size = 16, learning rate α = 0.01, initial learning rate momentum = 0.937, and weight decay coefficient = 0.0005.

### 3.2. Image Acquisition and Preprocessing

**Figure 11:**
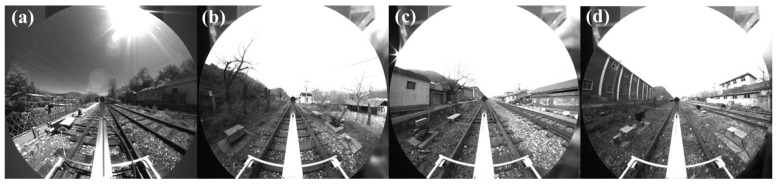
Panoramic images of different scenes at different times. (**a**) Summer noon. (**b**) Winter morning. (**c**) Spring afternoon. (**d**) Autumn morning.

Images were collected using our assembled inspection test vehicle; owing to the specific characteristics of the experimental environment, the equipment inside the tunnel was limited. Therefore, the main focus of the image collection process was on the equipment around the railway line. Immediately, the model was verified; it could be directly applied to detect equipment inside the tunnel. Because the scene was relatively simple, the collection time was divided by season, including spring, summer, autumn, and winter (with different surrounding scenes), as well as different times of the day (with different light intensities) as shown in Figure 11. The collected data were labeled using Labelimg.

**Figure 12:**
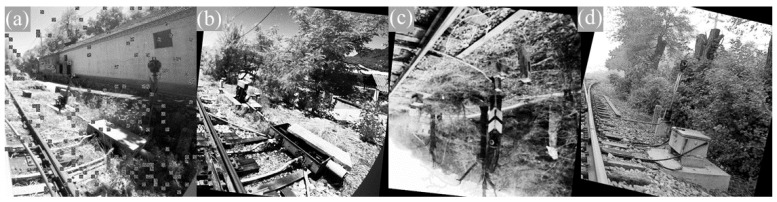
Images after cropping and augmentation. (**a**) Occluded and exposure changed. (**b**) Rotated and sharpened. (**c**) Flipped and blurred. (**d**) Rotated and contrast changed.

As the image size collected was 2048 × 2448, it was uniformly downsampled to 640 × 640 when input into the YOLOv5 network, which can result in a loss of a large amount of detailed information. Moreover, the two side parts of the image are the equipment distribution area, and the upper and lower parts are invalid blank areas. To address this issue, we considered cropping the collected images to preserve the image in the effective area. The resolution of the cropped image is 992 × 992. To further enrich the dataset and improve its generalization ability, we implemented data augmentation techniques to increase the amount of data as shown in Figure 12. The main operations include flip, rotation, image sharpening, Gaussian blur, contrast change, occlusion by adding black blocks, and exposure change. Meanwhile, semi-automatic image annotation was used to synchronize the enhancement of images and annotations. In addition to the existing Mosaic data augmentation, the mixup data augmentation was added to the model in this study. This method enhances the mixed-class images by blending images from different classes, which is an augmentation strategy independent of the data. It improves the generalization ability of small samples with less computational cost and has good applicability. The dataset was divided into training, validation, and testing sets in a ratio of 7:2:1. In total, approximately 5600 images were included, and the detection objects included 7 categories, such as signal lights, electrical boxes, switch machines, and derailleur machines.

### 3.3. Evaluation Metrics for Model Algorithms

In the experiments, Precision, Recall, mAP@0.5, mAP@0.5:0.95, and FPS (frames per second) in the test phase were used as evaluation metrics for the algorithm. Precision refers to the proportion of true positive predictions for a certain category among all positive predictions for that category. Recall represents the probability that a positive sample is predicted as positive among all actual positive samples. They are expressed as

(11) $$Pecision=\frac{TP}{TP+FP}$$

(12) $$Recall=\frac{TP}{TP+FN}$$

*TP* (true positive) represents the number of correctly detected objects, *FP* (false positive) represents the number of incorrectly detected objects, and *FN* (false negative) represents the number of undetected objects. Precision and recall are both important evaluation metrics for object detection. However, they are mutually restrictive and affect each other. Pursuing high precision causes low recall and vice versa. To comprehensively consider both metrics, AP (average precision) and mAP were used to measure the detection performance. AP is defined as the area under the PR curve, which is plotted with recall on the horizontal axis and precision on the vertical axis. mAP is calculated as follows:

(13) $$AP=\int_{0}^{1}P(R)dR$$

(14) $$mAP=\sum_{i=1}^{N}\frac{AP_{i}}{N}$$

*N* represents the number of detected categories; in this paper, *N* = 7. mAP@0.5 represents the average AP of all equipment categories at an IOU threshold of 0.5, which reflects the recognition ability of the model; mAP@0.5:0.95 represents the average value of each mAP when the IOU threshold ranges from 0.5 to 0.95 with a step of 0.05. mAP@0.5:0.95 requires a higher IOU threshold and reflects the localization effect and boundary regression ability. FPS represents the number of images detected per second; higher values indicate faster detection speed.

### 3.4. Experimental Results Analysis

#### 3.4.1. Comparison Experiments of Different Models

We compared the YOLOv5-CCFE model with YOLOv3, YOLOv4, Faster-RCNN, and original YOLOv5 to verify the accuracy and reliability of our proposed method. Considering the requirements of practical scenarios, we chose mAP@0.5, recall rate, precision, and FPS as evaluation indicators. We trained and validated these models on the railway equipment dataset, and the results are shown in Table 2. It can be observed that the YOLOv5 model in the detection algorithm has higher detection accuracy and recall rates compared to the two-stage FAST-RCNN and the one-stage YOLOv3 and YOLOv4 models. The FPS value of the single-stage detection algorithm YOLOv5 is 164, which is much higher than the two-stage detection algorithms and other single-stage detection algorithms in the table. In this article, the YOLOv5-CCFE model improves the precision and recall rate by 1.7% and 2.1%, respectively, compared to YOLOv5, and the mAP@0.5 is improved by 1.3%. The FPS reaches 158, which is slightly lower than the YOLOv5 model yet still meets the real-time requirements for railway tunnel equipment detection. Taking all factors into consideration, the performance of YOLOv5-CCFE is the optimal object detection algorithm.

According to Table 2, the YOLOv5 model in the detection algorithm has higher detection accuracy and recall rate compared to the two-stage FAST-RCNN and one-stage YOLOv3 and YOLOv4. The FPS value of the single-stage detection algorithm YOLOv5 is 164, significantly higher than the two-stage detection algorithm and other single-stage detection algorithms in the table. Our YOLOv5-CCFE model improves MAP@0.5 and recall rate by 1.3% and 2.1%, respectively, compared to YOLOv5. The FPS reaches 158, which is slightly lower than the YOLOv5 model but still meets the real-time detection requirements of railway tunnel equipment. Comprehensively, the performance of YOLOv5-CCFE is the optimal target detection algorithm in the table.

#### 3.4.2. Experiment Comparing Different Loss Functions

To verify the effectiveness of the Focal-EIOU loss function, we compared the experimental results of several types of loss functions, including CIOU, SIOU, Alpha-CIOU, EIOU, and Focal-EIOU, on the YOLOv5-CC (YOLOv5 + CARAFE + CBAMC3) algorithm. The results are shown in Table 3. The Focal-EIOU loss, compared to the original CIOU loss in YOLOv5, improves accuracy by 0.9% and increases the mAP@0.5 value by 0.4%. Compared to other IOU methods, it enhances both accuracy and recall rates. Furthermore, compared to using only the EIOU loss, it maintains the same accuracy while improving the recall rate by 1.2%.

**Figure 13:**
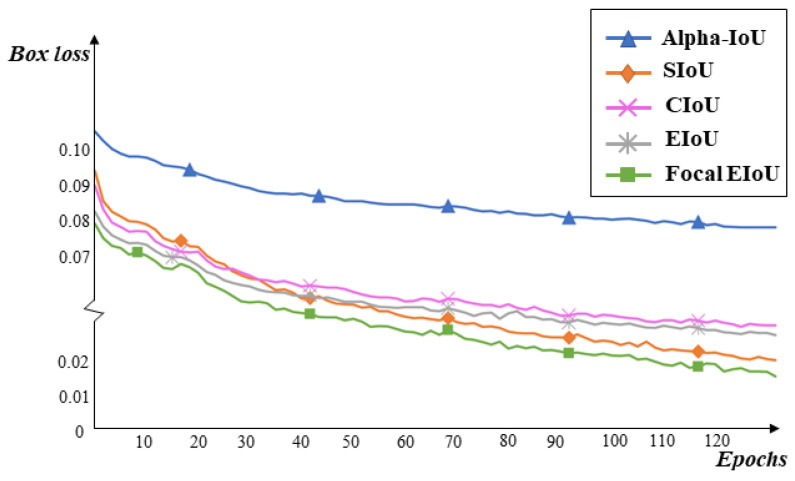
Regression box loss curve under different loss functions.

Figure 13 illustrates the impact of the five loss functions on the YOLOv5-CC algorithm. It can be observed that the regression box loss curve of the Focal-EIOU is the lowest, indicating that this loss has better regression accuracy and regression box loss compared to the other four loss functions.

#### 3.4.3. Visual Analysis

**Figure 14:**
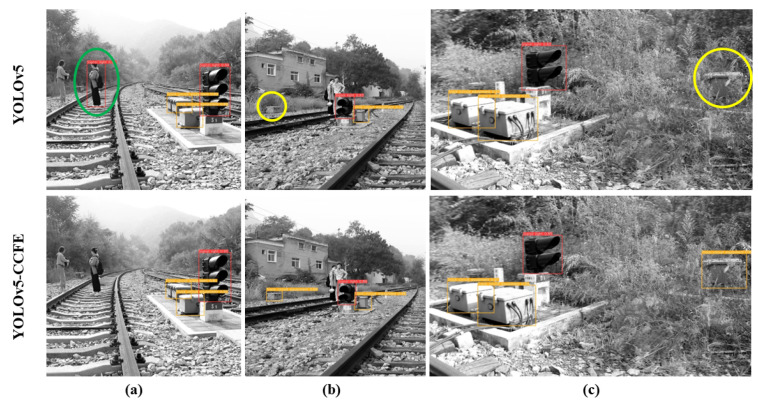
Image detection performance. (**a**) YOLOv5-CCFE figured out a false detection of a humanas a signal light of YOLOv5 in the green oval. (**b**) YOLOv5-CCFE detected a missed small track circuit equipment of YOLOv5 in the yellow circle. (**c**) YOLOv5-CCFE detected a missed track circuit equipment in the edge of YOLOv5 in the yellow circle.

To intuitively reflect the performance of the improved model, we selected representative cropped images and original uncropped panoramic images from the test set as test objects, and analyzed the device object detection results of the YOLOv5 algorithm and the YOLO-CCFE algorithm in different scenarios as shown in Figure 14.

**Figure 15:**
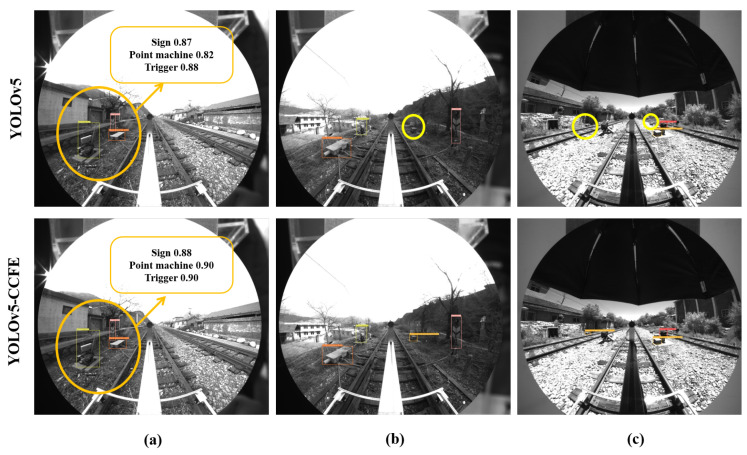
Original image detection performance of panoramic images. (**a**) YOLOv5-CCFE has higher mAP than YOLOv5. (**b**) YOLOv5-CCFE detected a missed small track circuit equipment in the yellow circle. (**c**) YOLOv5-CCFE detected 2 missed small track circuit equipment from far away in the yellow circle.

Comparing the results, we know that the YOLOv5-CCFE model can better avoid missing and false detections of targets. It can distinguish a person wearing black and white clothes mistakenly entering the signal light area (marked with a green ellipse in Figure 14a), and can effectively detect the less noticeable electrical equipment in the grass (marked with yellow ellipses in Figure 14b,c). Meanwhile, in the panoramic image detection as shown in Figure 15a, the confidence of various targets is improved slightly (marked with orange ellipse). For Figure 15c, although the YOLOv5-CCFE model also has omissions owing to the distance and small size of the target, it has advantages compared to the original YOLOv5.

To verify the robustness of the algorithm, we collected 300 images of railway equipment along the high-speed railway line by web crawlers for experimental verification. The accuracy recognition rate reached 84%. Some of the detection results are shown in the following figure.

**Figure 16:**
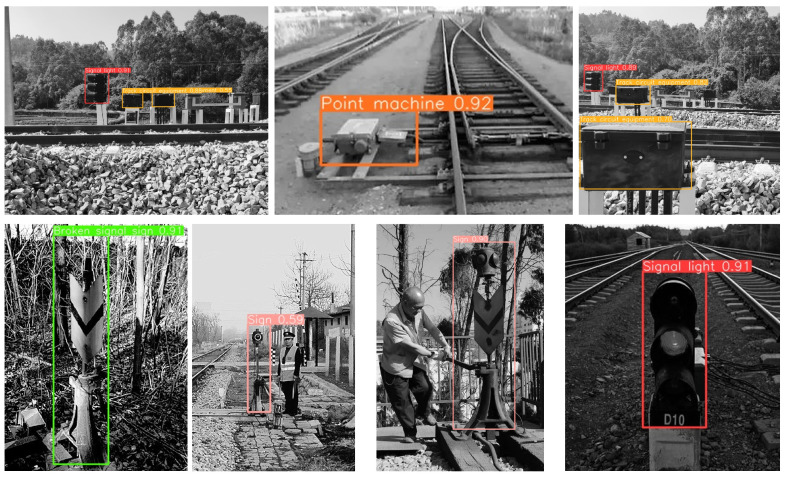
Model performance on crawler images.

From Figure 16, it can be visually observed that whether it is a black and white or color image, the model can basically recognize the devices arranged on both sides of the tunnel, which indicates good generalization of our model.

### 3.5. Ablation Experiment

To explore the effectiveness of our method and further validate the detection performance of our model, we gradually evaluated the improvement results by adding new components to the original YOLOv5 model. The experimental results are shown in Table 4.

**Figure 17:**
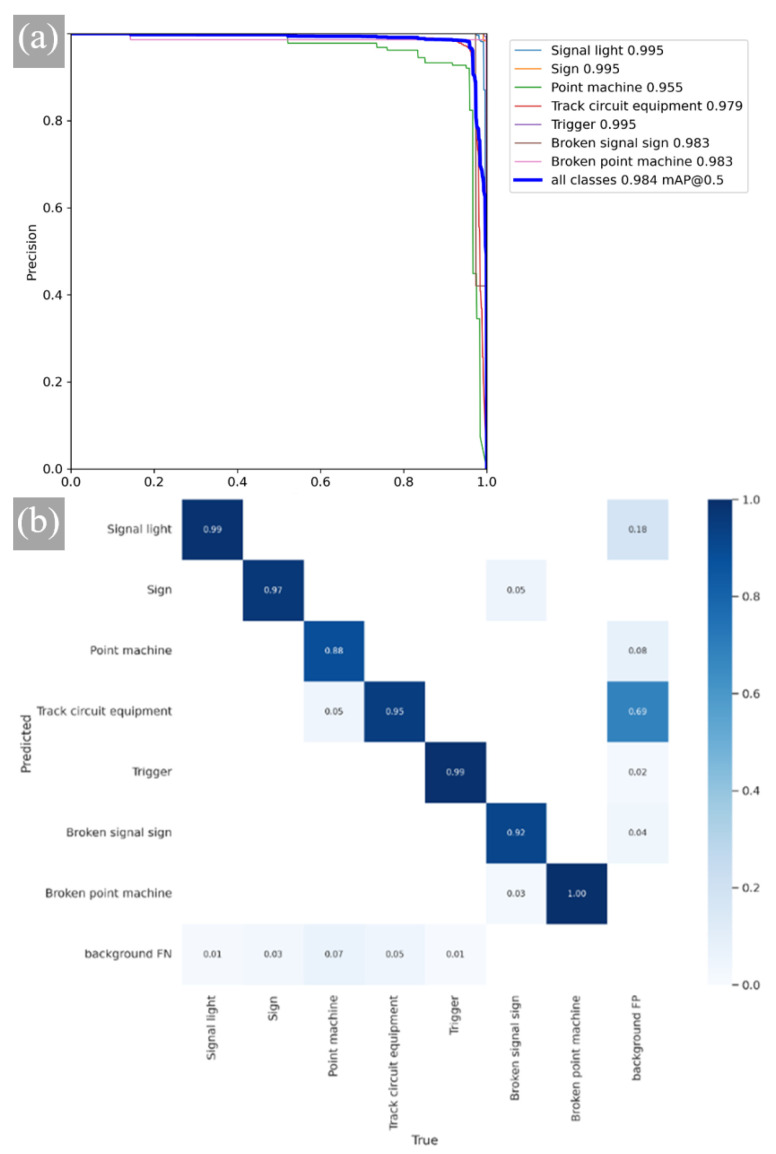
Analysis of YOLOv5-CCFE. (**a**) P-R curve. (**b**) Confusion matrix.

In this table, CARAFE represents the improved upsampling module in YOLOV5, CBAMC3 represents the introduction of the CBAM attention mechanism in C3, and Focal EIOU represents the modification of CIOU in the loss function. From Table 4, we can observe that after adding CARAFE, model mAP@0.5 is increased by 0.5%, while mAP@0.5:0.95 is increased by 1.6%, indicating an overall improvement in performance. By employing the CBAMC3 attention mechanism alone, the accuracy of the detection is improved by 0.5%, and mAP@0.5 is increased by 0.2%. After the introduction of Focal EIOU into the loss function, the recall ratio remains unchanged, but the precision ratio is improved. This indicates that Focal EIOU loss has a significant impact on the regression of detection frames. The final YOLOv5 CCFE model mAP@0.5 can reach 98.6%, which is 1.3% points higher than the original model, and MAP@0.5:0.95 is increased by 3.6% points. The RP curve of the final model is shown in Figure 17a.

The confusion matrix of the YOLOv5-CCFE model is shown in Figure 17b. Each column of the confusion matrix represents the instance prediction of a class, and each row represents an actual instance of a class. All the correct prediction results are on the diagonal. Through this matrix, two different types of confusion can be observed intuitively, which shows that the accurate prediction rate is more than 88%, indicating that the model has good recognition performance.

## 4. Conclusions

In order to meet the intelligent detection requirements of high-speed railway tunnel equipment, we introduce a hyperboloid catadioptric panoramic imaging system that can obtain real-time, wide-field imaging while shielding the high dynamic phenomena at the tunnel exit, facilitating the detection of the target equipment. We propose a detection model called YOLOv5-CCFE for high-speed railway equipment recognition. Firstly, in the feature fusion part of the standard YOLOv5, we introduce the content-aware reassembly of feature elements (CARAFE) module, which enhances the detection performance of small targets through fine-grained upsampling. Secondly, we incorporate the CBAM attention mechanism into the C3 module of the backbone network to focus more on device features. Finally, we replace the CIOU loss with the Focal-EIOU loss function, which improves the convergence speed and detection accuracy of the model. Comparative experiments show that the YOLOv5-CCFE model outperforms other models in both training and detection stages, achieving an mAP@0.5 value of 98.6% and an mAP@0.5:0.95 value of 68.9%. The FPS value is 158, indicating that the YOLOv5-CCFE technology is promising for the fast and automated inspection of devices along railway tunnel lines. Additionally, this inspection solution can also be applied to tasks such as detecting water seepage on tunnel walls, cracks in tunnel walls, and damage or detachment of rail fasteners.

Due to limitations in the conditions, it is currently not possible to conduct experiments in actual high-speed railway tunnels. Therefore, improvements can only be made based on common issues along railway tracks and within tunnels. For practical applications in railway maintenance, it is necessary to consider motion blur caused by high-speed movement. In the next step, research can be conducted on restoration algorithms for nonlinear space-invariant degraded blurry images to mitigate the impact of degraded images on target recognition. Additionally, it is possible to explore more lightweight object detection algorithm models by compressing the backbone network using methods such as pruning. This can improve the average detection speed, reduce model size, and consider algorithm deployment on embedded devices.

## Figures and Tables

**Table 1:** Experiment environment parameters.

Parameter	Experimental Environment
Operating System	Linux (Ubuntu18.04)
GPU	NVIDIA RTX3080Ti
Memory	64 GB
Python	3.8.11
Deep Learning Framework	Pytorch1.8.0/CUDA11.1

**Table 2:** Comparison results of performance among different algorithms.

Methods	mAP@0.5 (%)	Precision (%)	Recall (%)	FPS
Faster-RCNN	97	96.7	94.2	37
YOLOv3	93.5	92.9	91.6	59
YOLOv4	95.2	93.3	92.5	72
YOLOv5	97.3	96	94.8	164
YOLOv5-CCFE	98.6	97.7	96.9	158

**Table 3:** Comparison results of performance among different loss functions.

Loss	Precision (%)	Recall (%)	mAP@0.5 (%)
CIOU	96.8	97.3	98.2
SIOU	97.3	96.5	98.38
Alpha-CIOU	97.1	95.9	98.26
EIOU	97.7	95.8	97.9
Focal-EIOU	97.7	97.0	98.6

**Table 4:** Results of ablation experiments introducing different modules.

Algorithm	Module	Precision (%)	Recall (%)	mAP@0.5 (%)	mAP@0.5:0.95 (%)
YOLOv5	NONE	96.0	94.8	97.3	65.3
YOLOv5-CA	CARAFE	96.8	97.3	98.2	68.6
YOLOv5-CB	CBAMC3	96.5	94.6	97.5	67.3
YOLOv5-FE	Focal EIOU	96.3	94.8	97.7	67.9
YOLOv5-CCFE	ALL	97.7	97.0	98.6	68.9

## Data Availability

Not applicable.
